# Functional Networks of Reward and Punishment Processing and Their Molecular Profiles Predicting the Severity of Young Adult Drinking

**DOI:** 10.3390/brainsci14060610

**Published:** 2024-06-18

**Authors:** Yashuang Li, Lin Yang, Dongmei Hao, Yu Chen, Yiyao Ye-Lin, Chiang-Shan Ray Li, Guangfei Li

**Affiliations:** 1 Department of Biomedical Engineering, College of Chemistry and Life Science, Beijing University of Technology, 100 Pingleyuan, Beijing 100124, China; liyashuang666@emails.bjut.edu.cn (Y.L.);; 2 Beijing International Science and Technology Cooperation Base for Intelligent Physiological Measurement and Clinical Transformation, Beijing 100124, China; 3 BJUT-UPV Joint Research Laboratory in Biomedical Engineering, 46022 Valencia, Spain; 4 Department of Psychiatry, Yale University School of Medicine, New Haven, CT 06510, USAchiang-shan.li@yale.edu (C.-S.R.L.); 5 Centro de Investigación e Innovación en Bioingeniería, Universitat Politècnica de València, 46022 Valencia, Spain; 6 Department of Neuroscience, Yale University School of Medicine, New Haven, CT 06511, USA; 7 Interdepartmental Neuroscience Program, Yale University School of Medicine, New Haven, CT 06520, USA; 8 Wu Tsai Institute, Yale University, New Haven, CT 06511, USA

**Keywords:** alcohol use disorder, alcohol misuse, fMRI, connectome, neurotransmitter, receptor

## Abstract

Alcohol misuse is associated with altered punishment and reward processing. Here, we investigated neural network responses to reward and punishment and the molecular profiles of the connectivity features predicting alcohol use severity in young adults. We curated the Human Connectome Project data and employed connectome-based predictive modeling (CPM) to examine how functional connectivity (FC) features during wins and losses are associated with alcohol use severity, quantified by Semi-Structured Assessment for the Genetics of Alcoholism, in 981 young adults. We combined the CPM findings and the JuSpace toolbox to characterize the molecular profiles of the network connectivity features of alcohol use severity. The connectomics predicting alcohol use severity appeared specific, comprising less than 0.12% of all features, including medial frontal, motor/sensory, and cerebellum/brainstem networks during punishment processing and medial frontal, fronto-parietal, and motor/sensory networks during reward processing. Spatial correlation analyses showed that these networks were associated predominantly with serotonergic and GABAa signaling. To conclude, a distinct pattern of network connectivity predicted alcohol use severity in young adult drinkers. These “neural fingerprints” elucidate how alcohol misuse impacts the brain and provide evidence of new targets for future intervention.

## 1. Introduction

### 1.1. Reward and Punishment Processing in Alcohol Misuse

People may engage in drinking because of the positive (e.g., elevated sociability and physical relaxation) and/or the negative reinforcing (e.g., amelioration of negative emotions) effects of alcohol [1]. Distinguishing the mechanisms of positive and negative reinforcement helps in understanding of pathophysiology of craving [2] and how reward and punishment processing may interact with self-control in the etiological processes of alcohol misuse [3].

Brain imaging provides a venue for elucidating the neural bases of altered reward and punishment processing in alcohol misuse. For instance, higher levels of reward sensitivity may contribute to alcohol misuse, as did lower ventral striatal activity during reward anticipation [4]. A study using both the Human Connectome Project (HCP) and Genetic Neuroimaging (IMAGEN) datasets reported higher functional connectivity of the medial orbitofrontal cortex, a reward area, and impulsivity in heavy drinkers [5]. In contrast, in our recent study, we demonstrated that loss rather than win reactivity along with fronto-striatal responses during a gambling task captured individual variation in alcohol use severity in a neurotypical sample [6]. In a reward go/no-go task where participants needed to initiate action or to inhibit an action to win money and/or avoid monetary loss, heightened punishment sensitivity enhanced the neural activities of avoidance and, in turn, contributed to alcohol misuse [7]. This literature together highlights the relevance of psychological and neural processes of reward and punishment processing to the pathophysiology of alcohol misuse.

### 1.2. Functional Connectomics of Individual Traits and Neuropsychiatric Diagnoses

Connectome-based predictive modeling (CPM) is a data-driven approach for developing predictive models of brain behavior relationships and individual variability [8,9]. The model also allows “computational lesion” to reveal features that are important in prediction [10]. With CPM investigators extracted and summarized the most relevant connectivity features that were cross-validated in test data, and provided an estimate of the accuracy at which these features predict individual traits, including fluid intelligence [8], sustained attention [11], mean sleep duration [12], creative ability [13], and drug craving [14], or clinical conditions, including akinetic rigidity of Parkinson’s disease [15] and binge drinking [16]. For instance, with longitudinal multisite functional magnetic resonance imaging (fMRI) data, collected at ages 14 and 19, to assess whole-brain patterns of functional organization that predict alcohol use, a recent study identified networks associated with vulnerability for future and current problem drinking [14]. These studies of CPM characterized the systems-level neural markers that predict individual variation in health and illness.

### 1.3. Molecular Profiles of Functional Brain Networks

Despite a growing understanding of the systems-level mechanisms, little is known about cellular and molecular dysfunction underlying alcohol misuse in humans. Exploring how regional and circuit dysfunction of the brain may be related to neurotransmitter pathways could shed light on the pathophysiology and help develop treatment of alcohol use disorders (AUD). By associating cognitive, affective and/or sensorimotor processing with microscale neurotransmitter systems and cell type-specific transcriptional signatures would also provide a venue linking the findings from human studies and animal models to better understanding the mechanisms of alcohol misuse and many other neuropsychiatric disorders.

A useful approach to complementing systems-level findings is to investigate the molecular profiles of the neural networks identified by MRI. A tool for spatial correlation analyses of MRI data with nuclear imaging derived neurotransmitter maps, JuSpace (1.5) provides a biologically meaningful framework to this end [17] and offers novel insight into disease mechanisms and associated clinical features [18]. For instance, in a study of frontotemporal dementia, investigators associated the altered patterns of grey matter volume (GMV) with the distribution of dopamine and acetylcholine pathways in mutation carriers and showed more widespread involvement of dopamine, serotonin, glutamate and acetylcholine pathways in symptomatic individuals [19]. Another work focused on a population of unmedicated first-episode schizophrenia and reported GMV alterations in association with the expression of serotonin, dopamine, and gamma amino butyric acid (GABA) receptors and/or transporters [20]. Other studies combined MRI findings and JuSpace mapping to investigate the patterns of volumetric atrophy in link with changes in neurotransmitter pathways in Parkinson’s disease [21], multiple sclerosis [22], and primary progressive aphasia [18].

Investigators have also combined multimodal brain imaging with JuSpace to link systems and molecular findings. Using functional connectivity density mapping of resting-state fMRI data along with a Go/No-Go task (outside scanner), Cui and colleagues reported spatial correlation with the ability of behavioral inhibition in the patterns of expression of gene categories involving cellular and synaptic elements of the cerebral cortex and ion channel activity as well as the serotonergic system [23]. An earlier study employed both resting-state fMRI and structural MRI and associated changes in GMV and intrinsic connectivities with serotonergic, dopaminergic and μ-opioid receptor systems in heavy cannabis users [24]. Tang and colleagues investigated volumetric atrophy, glucose hypometabolism, and neurotransmitter distribution utilizing both MRI and positron emission tomography data in Rasmussen’s encephalitis [25]. Another study conducted meta-analytic co-activation analyses on lesion masks of individuals who acquired antisocial behaviors following their brain lesions and implicated multiple cortical and subcortical areas as well as the serotoninergic system [26].

Together, this literature supports the combination of MRI and JuSpace tools to associate systems and molecular profiles of cerebral dysfunction to better understand the pathophysiology of neurological and psychiatric conditions, including alcohol misuse.

### 1.4. The Present Study

Here, we applied CPM to fMRI data of a gambling task obtained from the Human Connectome Project (HCP). We characterized the connectivity features that predicted drinking severity. The gambling task involved win (reward) and loss (punishment) processing, and our previous study highlighted loss and fronto-striatal reactivities more than win reactivities in distinguishing individual severity of alcohol use [6]. In our first aim, we tested the hypotheses that whole-brain connectivity features during loss, as compared to win processing, would likewise better characterize alcohol use severity in this HCP sample of young adults. In our second aim, we combined the CPM findings and the JuSpace toolbox to investigate how the maps of connectivity features of alcohol use severity related to the molecular profiles and to better understand the molecular and cellular mechanisms underlying alcohol misuse.

## 2. Materials and Methods

### 2.1. Dataset and Demographics

With permission from the HCP [27] and as in our previous work [6,28,29,30,31,32], we employed the 1200 Subjects Release (S1200) data set, which includes 3T MR imaging and behavioral data collected of the gambling task from 1080 subjects. A total of 981 subjects (473 men, mean ± SD = 27.9 ± 3.6 years; 508 women, 29.6 ± 3.6 years) were included in this study, after exclusion of 99 with head movements >2 mm in translation or 2 degrees in rotation or for whom the images failed in registration to the template. All subjects were physically healthy with no severe neurodevelopmental, neuropsychiatric or neurological disorders. Because men and women differed significantly in age, age and sex were included as covariates in all analyses. The study was carried out in accordance with the latest version of the Declaration of Helsinki. HCP was approved by the Washington University Institutional Review Board (IRB #201204036).

### 2.2. Clinical Measures

We assessed drinking severity based on 15 intrinsically associated measures of alcohol consumption in the past year, as evaluated by the Semi-Structured Assessment for the Genetics of Alcoholism. With principal component analysis (PCA) to reduce the dimensionality of the 15 parameters, we used the first principal component or PC1 as a quantitative index of alcohol use severity. It should be noted that some of the 15 measures needed to be flipped in sign to reflect severity of drinking. PC1 had an eigenvalue = 7.42 and accounted for 49.47% of the variance of the data.

### 2.3. Neuroimaging Data Acquisition

Participants completed two runs of a gambling task each with 4 blocks (~3 m and 12 s each run)—2 each of reward and punishment, each with more win than loss trials, and more loss than win trials—and a fixation period (baseline, 15 s) between blocks [33,34,35]. The participants guessed whether the number of a mystery card (represented by a ‘?’ and ranging from 1 to 9) was larger or smaller than 5 by pressing a corresponding button. The feedback comprised a green up-pointing arrow for correct guess and $1 win, a red down-pointing arrow for $0.5 loss, or a gray double-headed arrow for a wash (mystery card number = 5). In reward blocks, 6 win trials were pseudo-randomly interleaved with either 1 neutral and 1 loss trial, 2 neutral trials, or 2 loss trials. In punishment blocks, 6 loss trials were interleaved with either 1 neutral and 1 win trial, 2 neutral trials, or 2 win trials. Preprocessing and plotting was conducted using SPM12 (7771) and the BioImage Suite (1.2.0), as described in our previous work [36,37,38] and the Supplementary Materials.

### 2.4. Functional Connectivity and Connectome-Based Predictive Modeling (CPM)

Whole-brain functional connectivity analyses were conducted using the BioImage suite. Network nodes were defined using the Shen 268-node brain atlas, which includes the cortex, subcortex and cerebellum. Task connectivity was calculated based on the ‘raw’ task time courses (punishment block only or reward block only). This involved computation of mean time courses for each of the 268 nodes (i.e., average time course of voxels within the node) for use in node-by-node pairwise Pearson’s correlations. R values of the 268 × 268 connectivity matrices represented the strength of connection between two individual nodes.

CPM was conducted using validated custom MATLAB (R2022b) scripts [9]. CPM took group connectivity matrices and behavioral data (in this case PC1) as inputs to generate a predictive model of PC1 from connectivity matrices, more details are described in the Supplementary Materials.

### 2.5. Correlation with Neurotransmitters

JuSpace (https://github.com/juryxy/JuSpace, accessed on 1 January 2021) allows for spatial correlation analyses between cross-modal neuroimaging data [17]. JuSpace consists of a group of Matlab functions together with PET imaging maps of various receptor and transporter systems, each with a distinct atlas. All receptor and transporter maps were derived of an average of 6 to 174 healthy volunteers and linearly rescaled to a minimum of 0 and a maximum of 100. To determine the neurochemical basis underlying the neural networks of drinking severity, we computed the spatial correlations of the SPM T maps derived from whole-brain regression each of “punishment-baseline” and “reward-baseline” against drinking PC1 and JuSpace maps of serotonin receptor (including 5-HT1a_1, 5-HT1a_2, 5-HT1b_1, 5-HT1b_2, 5-HT2a_1, 5-HT2a_2, 5-HT4); cannabinoid type I receptor (CB1); dopamine receptor (including D1,D2_1,D2_2); dopamine synthesis capacity receptor (FDOPA); gamma-aminobutric acid receptor (including GABAa_1, GABAa_2); mu opioid receptor (including MOR_1,MOR_2); metabotropic glutamate receptor (including mGluR5_1, mGluR5_2, mGluR5_3); dopamine transporter (DAT); noradrenaline transporter (NAT); serotonin transporter (including SERT_1, SERT_2, SERT_3); vesicular acetylcholine transporter (including VAChT_1, VAChT_2, VAChT_3). Pearson correlation coefficients between the T map and these neurotransmitter maps were calculated across the 119 brain regions of the neuromorphometrics atlas in JuSpace [17], excluding all white matter and cerebrospinal fluid regions. A Pearson’s correlation with *p* < 0.05 was considered significant.

## 3. Results

### 3.1. Drinking Severity PC1 Related Regional Activations

**Figure 1:**
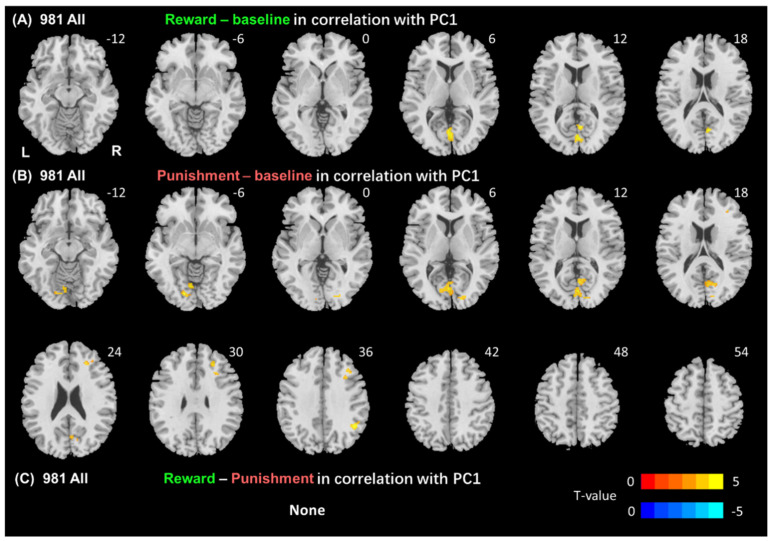
Regional responses in all subjects. Whole brain linear regression of the contrast (**A**) “reward-baseline”; (**B**) “punishment-baseline”; and (**C**) “reward-punishment”. L: left; R: right. Warm and cool colors show voxels for positive and negative correlation with PC1, respectively, etc.

We ran a whole-brain linear regression of the contrast “reward-baseline”, “punishment-baseline”, and “reward-punishment” with age and sex as covariates across all subjects (Figure 1, Table 1). We evaluated the results at voxel *p* < 0.001 uncorrected, combined with a cluster *p* < 0.05, FWE-corrected. However, please note that the T maps we used in conjunction with neurotransmitters’ atlases in JuSpace analysis were not thresholded (see below).

### 3.2. Predicting Drinking Severity PC1: Loss Processing

#### 3.2.1. CPM of Loss Processing

**Figure 2:**
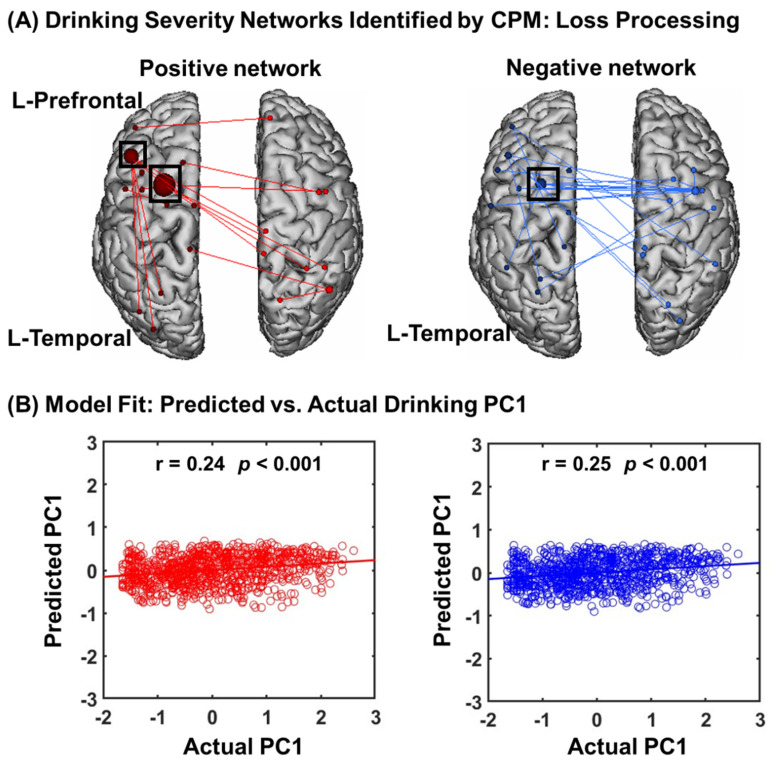
Macroscale network connectivities of loss processing in predicting drinking PC1. (**A**) shows positive (red) and negative (blue) networks in correlation with PC1. Larger spheres indicate nodes with more edges, and smaller spheres indicate fewer edges. For the positive network, higher edge weights (i.e., connectivity) predicted more severe drinking. For the negative network, lower edge weights predicted more severe drinking. (**B**) illustrates the correlation between actual (*x*-axis) and predicted (*y*-axis) drinking PC1 values generated using CPM.

In CPM of loss processing, the connectomics of positive and negative networks combined successfully predicted drinking severity PC1 (r = 0.25, *p* = 0.001, Figure 2A), as did connectivity within the positive (r = 0.24, *p* = 0.001) and negative (r = 0.25, *p* = 0.001) networks, separately (Figure 2B).

In addition to leave-one-out cross-validation, we also used 5-fold cross-validation. As shown in Supplementary Figure S1, negative (r = 0.12, *p* < 0.001) but not positive (r = 0.07, *p* = 0.223) networks significantly predicted PC1. It thus appears that, for loss processing, negative networks are more robust in predicting drinking severity.

#### 3.2.2. Network Anatomy of Loss Processing

**Figure 3:**
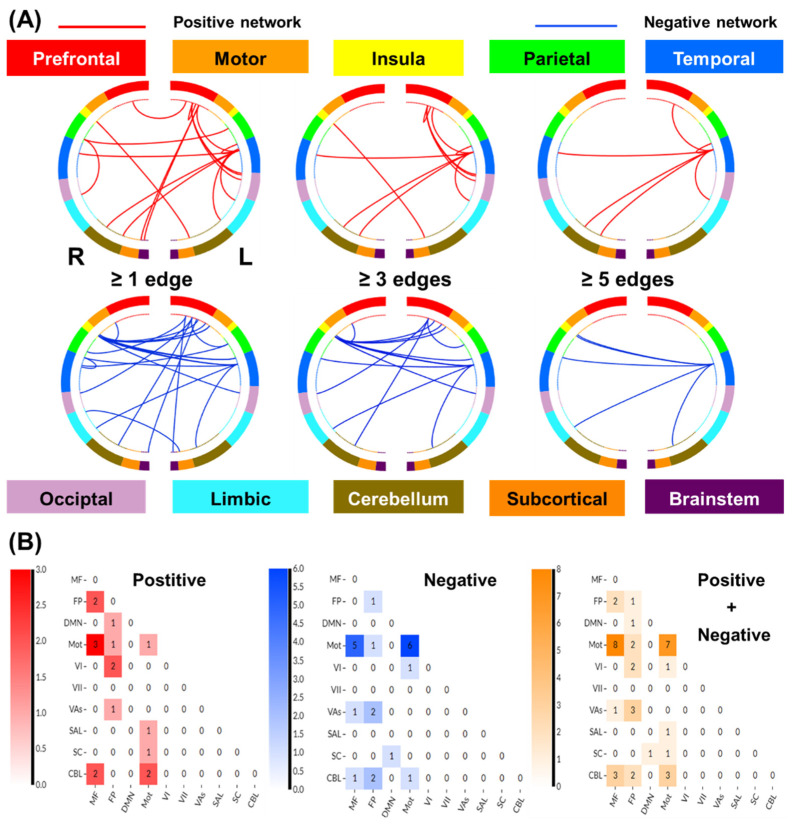
Macroscale network connectivities of loss processing in predicting drinking PC1. (**A**) Positive and negative networks summarized by connectivity between macroscale brain regions during punishment blocks. From the top, brain regions are presented in approximate anatomical order, such that longer-range connections are represented by longer lines. (**B**) Positive and negative and the sum of positive and negative networks. Cells represent the total number of edges connecting nodes within and between each network, with a higher number indicating a greater number of edges. Abbreviations: MF, medial frontal; FP, fronto-parietal; DMN, default mode; Mot, motor/sensory; VI, visual a; VII, visual b; Vas, visual assoc; SAL, salience; SC, subcortical; CBL, cerebellum/brainstem.

We summarized positive and negative networks based on the connectivities between macroscale brain regions (Figure 3A). Note that brain regions are presented in approximate anatomical order, such that longer-range connections are represented by longer lines. The network anatomies were complex and included connections between the frontal, temporal, parietal lobes, cerebellum, and brainstem. Despite this complexity, these networks appeared to be quite specific, with positive and negative networks together including only 39 edges (17 positive and 22 negative), or less than 0.11% of all possible connections correlated with drinking severity PC1. The highest-degree nodes (i.e., nodes with the most connections) for the positive network included a temporal node with connections to prefrontal, parietal, limbic, cerebellar, and other temporal nodes and prefrontal nodes with connections to the occipital and temporal cortex. The highest-degree nodes for the negative network also included a temporal node with connections to the insula and limbic nodes, as well as connections to the cerebellar and other temporal nodes. Both networks included short- and long-range connections. All edges showing a significant correlation with PC1 are shown in Supplementary Table S1.

To characterize the networks of loss processing, we summarized the patterns of connectivity based on the number of connections within and between canonical neural networks [40] (Figure 3B). By definition, positive and negative networks do not contain overlapping connections; a single edge cannot be part of both a positive and a negative network. However, positive and negative networks included connections within and between similar large-scale canonical neural networks. The positive networks included relatively more connections, involving the medial frontal, frontoparietal, and motor/sensory networks. The negative networks included relatively more connections between motor/sensory and medial frontal; between visual association and fronto-parietal; and between fronto-parietal and cerebellar networks. The positive network was further characterized by more within-network connections across medial frontal and fronto-parietal and motor/sensory and cerebellar networks, whereas the negative network included more within-network connections for motor/sensory and medial frontal networks.

#### 3.2.3. Neurotransmitters Associated with Network Predictors of Alcohol Use Severity: Loss Processing

**Figure 4:**
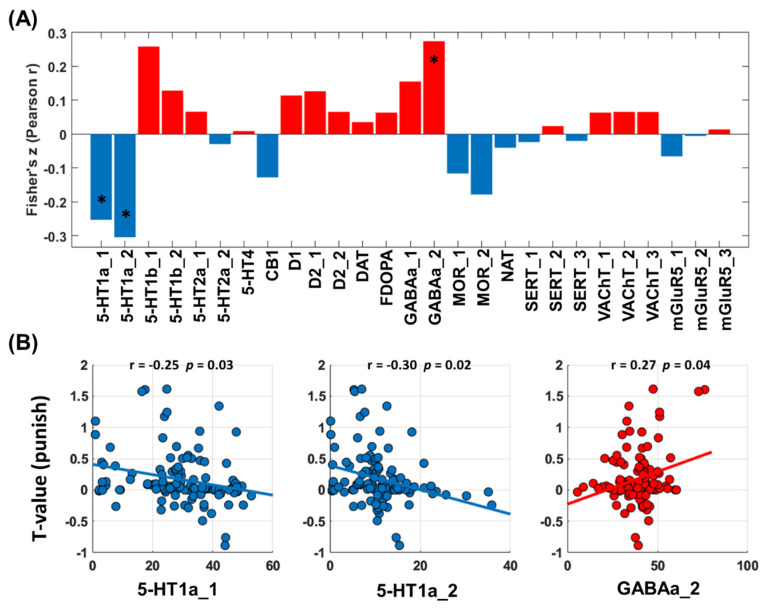
Correlations between T values of whole-brain regression of the contrast “punishment-baseline” against PC1 and neurotransmitter distribution maps. (**A**) T maps and neurotransmitter correlation analysis results (red/blue each represents positive/negative Pearson’s r). (**B**) Transporter or receptor systems that are significantly associated with T maps. Abbreviations: 5-HT,5-hydroxytryptamine (serotonin); CB1, cannabinoid type 1; D, dopamine receptor; DAT, dopamine transporter; FDOPA, fluorodopa, an analog of L-DOPA to assess the nigrostriatal dopamine system; GABAa, gamma-aminobutyric acid a; MOR, mu opioid receptor; NAT, noradrenaline transporter; SERT, serotonin transporter; VAChT, vesicular acetylcholine transporter; mGluR5, metabotropic glutamate type 5. *: *p* < 0.05.

Cross-region spatial correlation analyses revealed a significant link between the network correlates of alcohol severity PC1 and serotonergic (5-HT) and GABAergic densities (Figure 4A). The 5-HT system involved specifically the 5-HT1a receptors, and the GABAergic system involved the GABAa2 receptors (Figure 4B).

### 3.3. Predicting Drinking Severity: Win Processing

#### 3.3.1. CPM of Win Processing

**Figure 5:**
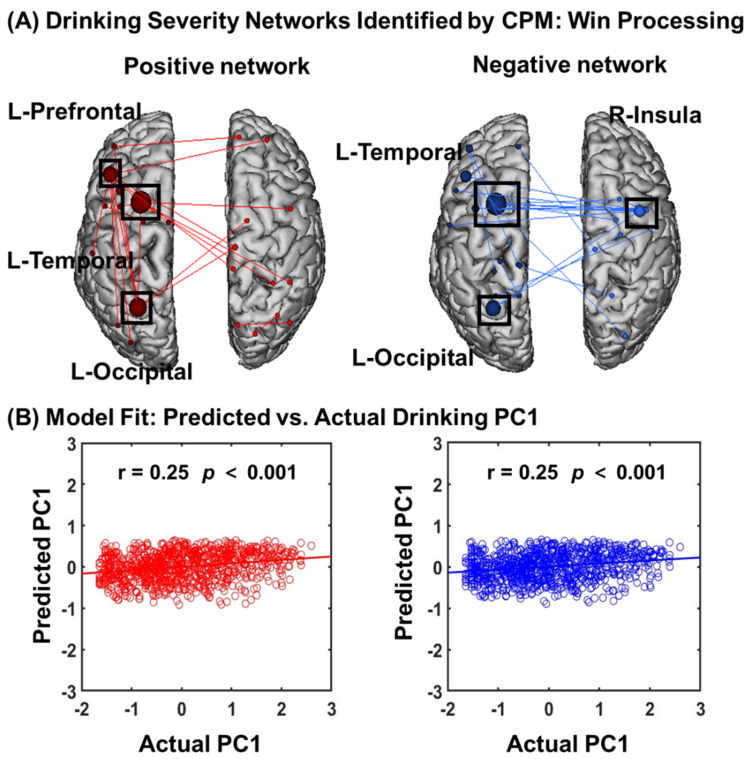
Macroscale network connectivities of win processing in predicting drinking PC1. (**A**) shows positive (red) and negative (blue) networks. For the positive network, higher edge weights (i.e., connectivity) predict more severe drinking. For the negative network, lower edge weights predict more severe drinking. Larger spheres indicate nodes with more edges, and smaller spheres indicate fewer edges. (**B**) illustrates the correlation between actual (*x*-axis) and predicted (*y*-axis) drinking severity values generated using CPM.

In CPM of win processing in the gambling task (Figure 5A), the overall model (positive and negative networks combined) successfully predicted drinking severity PC1 (r = 0.25, *p* = 0.001), as did connectivity within the positive (r = 0.25, *p* = 0.001) and negative (r = 0.25, *p* = 0.001) networks separately (Figure 5B).

In addition to leave-one-out cross-validation, we also used 5-fold cross-validation. As shown in Supplementary Figure S2, both positive and negative networks significantly predicted PC1, though the strength of prediction was weaker (positive network: r = 0.15, *p* < 0.001; negative network: r = 0.13, *p* < 0.001).

#### 3.3.2. Network Anatomy of Win Processing

**Figure 6:**
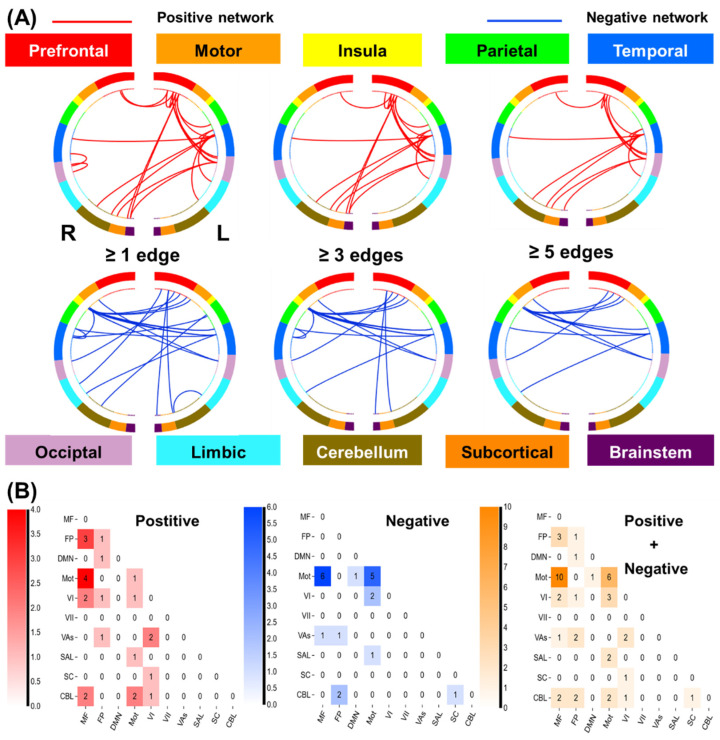
Macroscale network connectivities of win processing in predicting drinking PC1. (**A**) Positive and negative networks summarized by connectivity between macroscale brain regions during punishment blocks. From the top, brain regions are presented in approximate anatomical order, such that longer-range connections are represented by longer lines. (**B**) Positive and negative and the sum of positive and negative networks. Cells represent the total number of edges connecting nodes within and between each network, with a higher number indicating a greater number of edges. Abbreviations: MF, medial frontal; FP, frontal parietal; DMN, default mode; Mot, motor/sensory; VI, visual a; VII, visual b; Vas, visual assoc; SAL, salience; SC, subcortical; CBL, cerebellum/brainstem.

Network anatomies for both networks were complex and included connections between the frontal, temporal, parietal, cerebellum, and brainstem lobes (Figure 6A). However, the spatial extent of both positive and negative networks together included only 44 edges (24 positive, 20 negative), or less than 0.12% of all possible connections correlated with drinking severity PC1. The highest-degree nodes (i.e., nodes with the most connections) for the positive network included a prefrontal node with connections to temporal, occipital, and limbic nodes and a temporal node with connections to cerebellar, subcortical, and other temporal nodes. The highest-degree nodes for the negative network also included a temporal node with connections to the insula, cerebellar, and brainstem nodes, as well as a temporal node with connections to the insula, limbic, and other temporal nodes. Both networks included short- and long-range connections. Compared to loss processing, win processing involved more functional connections in the prefrontal, subcortical, and brainstem networks. All edges showing a significant correlation with PC1 are shown in Supplementary Table S2.

Likewise, we summarized the connectivities based on the number of connections within and between canonical neural networks (e.g., frontoparietal, motor/sensory) for the positive and negative networks (Figure 6B). The positive networks included relatively more connections of medial frontal, fronto-parietal, and motor/sensory networks. The negative network included relatively more connections between motor/sensory and medial frontal. The positive network was further characterized by more within-network connections across medial frontal and fronto-parietal and motor/sensory and cerebellar networks, whereas the negative network included more within-network connections for motor/sensory and medial frontal networks.

#### 3.3.3. Neurotransmitters Associated with Network Predictors of Alcohol Use Severity: Win Processing

**Figure 7:**
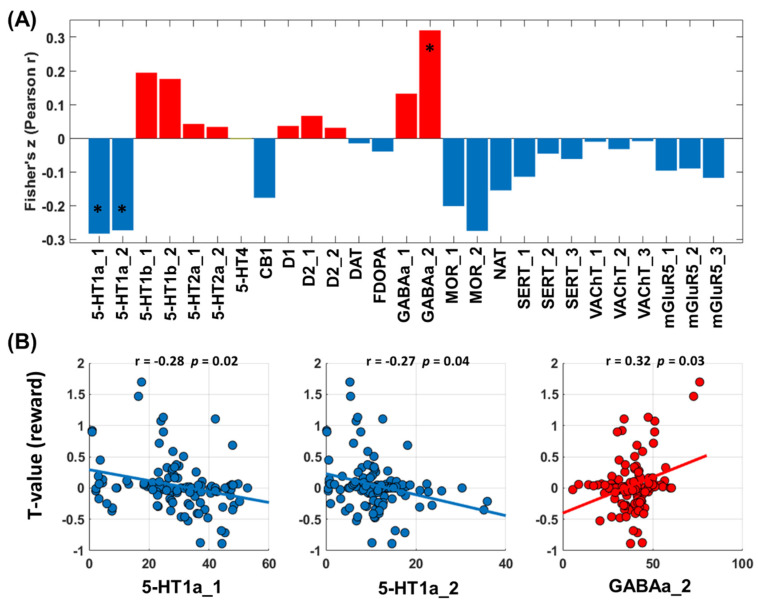
Correlations between T values of whole-brain regression of the contrast “reward-baseline” against PC1 and neurotransmitter distribution maps. (**A**) T maps and neurotransmitter correlation analysis results (red/blue each represents positive/negative Pearson’s r). (**B**) Transporter or receptor systems that are significantly associated with T maps. Abbreviations: 5-HT,5-hydroxytryptamine (serotonin); CB1, cannabinoid type 1; D, dopamine receptor; DAT, dopamine transporter; FDOPA, fluorodopa, an analog of L-DOPA to assess the nigrostriatal dopamine system; GABAa, gamma-aminobutyric acid a; MOR, mu opioid receptor; NAT, noradrenaline transporter; SERT, serotonin transporter; VAChT, vesicular acetylcholine transporter; mGluR5, metabotropic glutamate type 5. *: *p* < 0.05.

As with loss processing, cross-region spatial correlation analyses revealed a significant link between the network correlates of alcohol severity PC1 and serotonergic (5-HT) and GABAergic densities during win processing (Figure 7A). The 5-HT system involved specifically the 5-HT1a receptors, and the GABAergic system involved the GABAa2 receptors (Figure 7B).

## 4. Discussion

In this study, we demonstrated the utility of a connectome-based machine learning approach in predicting alcohol use severity using whole-brain functional connectivities in a gambling task. The connectomics predominantly involving medial frontal, motor/sensory, and cerebellum/brainstem networks during punishment processing and those involving medial frontal and fronto-parietal, motor/sensory networks during reward processing predicted drinking severity. Further, with JuSpace, we demonstrated in spatial correlation analyses that these networks were associated specifically with 5-HT1a and GABAa signaling. Together, these findings highlight connectivity markers of alcohol use severity and the molecular profiles of these markers in young adults.

### 4.1. Connectivity Features That Predict Drinking Severity

With connectivity features for both loss and win processing during gambling, CPM successfully predicted drinking severity. Although the specific features varied, the positive networks included connections amongst the medial frontal, motor/sensory, and fronto-parietal networks, and the negative networks included connections between motor/sensory and medial frontal; between visual association and fronto-parietal; and between fronto-parietal and cerebellar networks for both loss and win processing. In contrast, whereas loss processing involved positive temporal, prefrontal, and subcortical network connections and negative temporal and limbic, including insula, connections, win processing involved positive network connections between temporal and limbic, between prefrontal and occipital, and negative network connections amongst insula, prefrontal, and temporal cortices.

These findings are broadly consistent with reports of dysfunctional activation of the network nodes during psychological processes of importance to habitual drinking. For instance, an earlier work associated duration of alcohol use with lower activation in the right inferior frontal gyrus extending to superior temporal gyrus during inhibitory control, which was not observed for age-related changes in nondrinkers [41]. Another study demonstrated cue-craving circuits that involved connectivities with the frontal, parietal, and temporal brain regions in AUD participants vs. controls [42]. Whereas it is challenging to relate these activity or connectivity findings directly to the connectomics features identified in the current study, it appears that the same brain regions may participate in various psychological processes that conduce to alcohol consumption via their wide network connectivities.

On the other hand, these connectivity results contrasted with our earlier findings that loss but not win reactivities distinguished individual variation in drinking [6] and suggested connectivity features as additional neural markers of alcohol use severity. Secondly, shared connectivity features of loss and win processing suggest the potential role of saliency circuit dysfunction in alcohol misuse. The current findings can also be discussed with earlier CPM studies of drinking or other substance use. For instance, Rapuano and colleagues demonstrated that both resting and reward-related connectomics in developing brains may predict risk for substance use, as reflected in, e.g., substance-related behavioral measures and family history of drug use [43]. Another study applied CPM to the International Neuroimaging Data-sharing Initiative database and identified functional connectivity from the DMN to the sensorimotor, opercular, and occipital networks in predicting empathy in healthy subjects but not in abstinent drinkers, suggesting individual variation in the degree and pattern of network disruption as a result of chronic alcohol exposure [44]. Other studies of CPM reported connectivities of the motor/sensory, salience, and executive control networks during cue exposure in the prediction of dependence severity in male smokers [45], stronger motor/sensory within-network connectivity, and reduced connectivity between the motor/sensory and medial frontal, default mode, and frontoparietal networks in distinguishing opioid from other substance use disorders [46]. 

An important question concerns whether the connectomics features would predict future drinking, as was successfully demonstrated for reward-related fMRI data in a treatment study of cocaine use disorders [47]. In a magnetoencephalography (MEG) study of adolescents described the relationship between functional Connectivity (FC) during an inhibitory control (IC) task and development of heavy drinking over a two-year period. The results showed that higher beta-band FC in the prefrontal and temporal regions at baseline predicted higher levels of future alcohol consumption. Further, greater future alcohol consumption was associated with more severe reduction in the same FC’s [48]. Another study showed that greater severity of binge drinking in college students was negatively associated with connectivity between the DMN and ventral attention network, although CPM failed to identify a generalizable predictive model of longitudinal changes in connectivity edges over follow-up of two years [16]. It remains to be seen whether the current network markers predict future alcohol use and identify individuals at risk for AUD.

### 4.2. Molecular Profiles of the Connectivity Networks

We examined the molecular profiles of the network markers, and the results highlighted the role of the GABAergic and serotonergic signaling in alcohol use severity. This finding is consistent with a large body of clinical and preclinical research implicating the GABAergic and serotonergic systems in alcohol misuse [49,50,51,52].

Alcohol mimics the activity of GABA by binding to GABA receptors and inhibits neuronal activities, leading to widespread suppression of brain function. Other studies implicated GABAergic systems in dependent drinking. For instance, injection of muscimol, a GABAa receptor agonist, in the ventral tegmental area (VTA) elevated voluntary drinking in alcohol-preferring AA rats [53]. In a study quantifying the levels of transcript expression of GABAa receptor mRNA in postmortem brain tissues, those with a diagnosis of AUD who died of cirrhotic liver disease (suggesting higher level of alcohol use) as compared to controls had significantly higher expression of the transcripts in dorsolateral prefrontal and primary motor cortices [54].

Preclinical work demonstrated the roles of 5-HT1A-dependent regulation of binge drinking from short- to longer-term alcohol exposure that involves the dentate gyrus of the hippocampus [55]. In a drinking-in-the-dark (DID) paradigm to model chronic binge-like voluntary alcohol consumption in mice, selective partial activation of 5-HT1A receptors by tandospirone (5-HT1A partial agonist) prevented alcohol withdrawal-induced anxiety-related behavior and binge-like ethanol intake. Further, DID-elicited deficits in neurogenesis in the dorsal hippocampus were reversed by chronic treatment with tandospirone [56]. An earlier study characterized the opposing effects of stimulation of somatodendritic 5-HT1A receptors at lower doses and postsynaptic 5-HT1A receptors at higher doses on alcohol intake in Long–Evans rats [57]. In PET imaging of 5-HT1A receptor binding in alcohol-naïve rhesus, the binding potential increased in the raphe nuclei (vs. the cerebellum as a reference region) after chronic ethanol self-administration. Further, baseline 5-HT1A binding in the raphe nuclei showed a positive correlation with average daily ethanol self-administration [58]. In a large sample of adolescents, 5-HTTLPR low-activity allele carriers exposed to higher levels of family conflict were more likely to engage in alcohol misuse than non-carriers [59].

Together, the current findings are consistent with this previous body of work implicating GABAergic and serotonergic signaling in alcohol misuse. However, many other studies implicated the glutamatergic [60,61,62], dopaminergic [63], cannabinoid [64], and GABAb [65] signaling in alcohol misuse. Some of these systems which have yet to be covered by the JuSpace toolbox need to be further investigated along with the network markers of alcohol use severity.

### 4.3. Speculation on Research and Clinical Implications

Alcohol impacts multiple neurotransmitters, including dopaminergic, serotonergic, glutamatergic, GABAergic, and opioid systems, in the brain. These systems interact to support brain functions and the effects of alcohol on brain functions. For instance, alcohol elevates dopaminergic activity in the VTA circuit through decreasing the suppressant influences of GABAergic inputs [66,67,68]. Thus, central GABAergic neurotransmission is linked to mesolimbic dopaminergic neurotransmission during rewarding processes and may represent a pharmacotherapeutic target for the treatment of AUD [69]. Further, dopamine D2 receptors in the striatum are primarily localized in GABAergic neurons, providing more evidence of GABAergic involvement in dopaminergic dysfunction and alcohol misuse [70]. A study of social drinkers associated GABA receptor α2 subunit gene (GABRA2) with subjective effects of alcohol, pleasant or unpleasant, suggesting that GABRA2 may play a role in the risk of AUDs by moderating the subjective effects of alcohol [71]. These examples, along with the current findings implicating GABAergic but not directly dopaminergic neurotransmission in the severity of drinking, suggest the importance of investigating multiple neurotransmitters in understanding the psychological and systems mechanisms of alcohol misuse and in researching interventions for AUD.

Depression is highly comorbid with alcohol misuse. While the efficacy of many antidepressants is supported by monoaminergic mechanisms, new rapid-acting agents target glutamatergic and GABAergic systems [72,73]. Thus, human genetics and imaging in combination with preclinical studies may help in identifying candidate circuits and neurotransmitter systems that are altered in relation to depression, alcohol misuse, or both. These findings would be critical to characterizing individual differences in the pathophysiology of comorbidity and developing novel antidepressants tailored to specific patient populations with distinctive phenotypes of molecular and circuit dysfunction [74]. Notably, evaluating changes in neurotransmitter systems would typically require molecular imaging [75,76,77] or fMRI of specific neurotransmitter circuits [38,78,79,80,81]. Along with earlier studies, the current work highlights the potential of combining fMRI and JuSpace data to link the systems and molecular processes altered in neuropathology. These new imaging biomarkers will certainly evolve and, as the dataset expands, offer new insights into the treatment of mental illnesses.

Methodologically, large-scale functional or structural brain connectivity can be modeled as a network, or graph. Thus, many graph-theoretic approaches can be used to identify and validate connections associated with a diagnostic status in case-control studies, individual cognitive and behavioral measures, or treatment responses. For instance, the network-based statistic (NBS) controls the family-wise error rate and combines/maps the nodes and connections of the graphs across multiple templates, thereby achieving data consistency across studies [82]. In particular, using multi-scale brain templates helps integrate patterns of functional connectivity across different spatial scales and may further our knowledge of brain network function and dysfunction and their molecular underpinnings [83].

### 4.4. Limitations and Conclusions

A few limitations need to be considered for this study. First, the networks identified from CPM included nodes from Shen’s atlas of 268 ROIs, whereas the neurotransmitter maps were of 119 brain regions. This discrepancy may have accounted for the missing links with other neurotransmitter systems. Second, the HCP gambling task contained a brief baseline (only 15 s) in between the winning and losing blocks, where participants were just fixating on the screen. Because of the short duration of the baseline and the neural dynamics that likely carried over from winning/losing blocks to the baseline, we did not consider the baseline in computing the connectivity matrices. Third, the HCP data represent a non-clinical sample; it thus remains to be seen whether the current findings can be generalized to alcohol use disorders. Fourth, alcohol misuse involves many other comorbidities, including smoking and internet gaming disorder [84], as well as depression and anxiety [85,86], that may implicate changes in cerebral connectomics. Although a recent study of a large cohort of adolescents highlighted resting-state connectivity features across the majority of networks are more strongly predictive of drinking than smoking [87], it remains to be seen whether the connectivity features identified here are specific to alcohol use severity. Finally, many large-scale studies have characterized the gray matter volumetric and thickness and white matter integrity markers of alcohol misuse [88,89,90,91]. Future studies can examine how functional and structural markers compare in the effect size of prediction and whether the combination of multimodal imaging makers improves the performance of CPM.

In conclusion, this study demonstrates that patterns of whole-brain connectivity during loss and win processing can predict drinking severity, and these connectivity markers are associated with serotonergic and GABAa signaling. These findings demonstrate that individual differences in connectivity within large-scale neural networks implicated in punishment and reward responses contribute to the severity of alcohol misuse outcomes. As such, these “neural fingerprints” may represent appropriate targets for future intervention efforts.

## Figures and Tables

**Table 1:** Regional responses of “reward-baseline”, “punishment-baseline”, and “reward-punishment” in correlation with drinking PC1 of all subjects.

Region	Cluster Size	Peak Voxel (T)	Cluster FWE *p*-Value	MNI Coordinates (mm)		
				X	Y	Z
“reward-baseline”						
Calcarine	228	4.20	0.000	4	−64	14
“punishment-baseline”						
L lingual	138	4.83	0.001	−16	−80	−8
R angular	76	4.81	0.031	44	−52	36
R frontal middle cortex	69	4.77	0.046	24	38	26
R calcarine	77	4.40	0.029	14	−88	10
R calcarine	398	4.25	0.000	4	−64	14
R frontal middle cortex	71	4.18	0.041	30	24	32
“reward-punishment”						
None						

Note: Brain regions were identified by reference to the Automated Anatomic Labeling or AAL Atlas [39]. L: left; R: right.

## Data Availability

All data needed to evaluate the conclusions in the paper are shown in the paper and the supplement. All raw data supporting the analyses and findings of this study are available from HCP.

## Supplementary Materials

The following supporting information can be downloaded at: https://www.mdpi.com/article/10.3390/brainsci14060610/s1, Section S1: Supplementary Methods; Figure S1: Macroscale network connectivities of loss processing in predicting drinking PC1. The correlation between actual (*x*-axis) and predicted (*y*-axis) drinking PC1 values generated using CPM with a five-fold cross-validation.; Figure S2: Macroscale network connectivities of win processing in predicting drinking PC1. The correlation between actual (*x*-axis) and predicted (*y*-axis) drinking PC1 values generated using CPM with a five-fold cross-validation; Table S1. The correlation between FC’s of punishment processing and drinking severity PC1; Table S2. The correlation between FC’s of reward processing and drinking severity PC1. Refs. [92,93,94] are cited in Supplementary Materials.

## References

[1] Koob G.F., Volkow N.D. (2016). Neurobiology of addiction: A neurocircuitry analysis. Lancet Psychiatry.

[2] Chen Y., Li C.-S.R. (2023). Appetitive and aversive cue reactivities differentiate neural subtypes of alcohol drinkers. Addict. Neurosci..

[3] Kahn R.E., Chiu P.H., Deater-Deckard K., Hochgraf A.K., King-Casas B., Kim-Spoon J. (2018). The Interaction Between Punishment Sensitivity and Effortful Control for Emerging Adults’ Substance Use Behaviors. Subst. Use Misuse.

[4] Moreno Padilla M., O’Halloran L., Bennett M., Cao Z., Whelan R. (2017). Impulsivity and Reward Processing Endophenotypes in Youth Alcohol Misuse. Curr. Addict. Rep..

[5] Cheng W., Rolls E.T., Robbins T.W., Gong W., Liu Z., Lv W., Du J., Wen H., Ma L., Quinlan E.B. (2019). Decreased brain connectivity in smoking contrasts with increased connectivity in drinking. Elife.

[6] Li G., Chen Y., Chaudhary S., Tang X., Li C.-S.R. (2022). Loss and Frontal Striatal Reactivities Characterize Alcohol Use Severity and Rule-Breaking Behavior in Young Adult Drinkers. Biol. Psychiatry Cogn. Neurosci. Neuroimaging.

[7] Le T.M., Zhornitsky S., Wang W., Ide J., Zhang S., Li C.R. (2019). Posterior Cingulate Cortical Response to Active Avoidance Mediates the Relationship between Punishment Sensitivity and Problem Drinking. J. Neurosci..

[8] Finn E.S., Shen X., Scheinost D., Rosenberg M.D., Huang J., Chun M.M., Papademetris X., Constable R.T. (2015). Functional connectome fingerprinting: Identifying individuals using patterns of brain connectivity. Nat. Neurosci..

[9] Shen X., Finn E.S., Scheinost D., Rosenberg M.D., Chun M.M., Papademetris X., Constable R.T. (2017). Using connectome-based predictive modeling to predict individual behavior from brain connectivity. Nat. Protoc..

[10] Feng C., Yuan J., Geng H., Gu R., Zhou H., Wu X., Luo Y. (2018). Individualized prediction of trait narcissism from whole-brain resting-state functional connectivity. Hum. Brain Mapp..

[11] Rosenberg M.D., Finn E.S., Scheinost D., Papademetris X., Shen X., Constable R.T., Chun M.M. (2016). A neuromarker of sustained attention from whole-brain functional connectivity. Nat. Neurosci..

[12] Mummaneni A., Kardan O., Stier A.J., Chamberlain T.A., Chao A.F., Berman M.G., Rosenberg M.D. (2023). Functional brain connectivity predicts sleep duration in youth and adults. Hum. Brain Mapp..

[13] Beaty R.E., Kenett Y.N., Christensen A.P., Rosenberg M.D., Benedek M., Chen Q., Fink A., Qiu J., Kwapil T.R., Kane M.J. (2018). Robust prediction of individual creative ability from brain functional connectivity. Proc. Natl. Acad. Sci. USA.

[14] Antons S., Yip S.W., Lacadie C.M., Dadashkarimi J., Scheinost D., Brand M., Potenza M.N. (2023). Connectome-based prediction of craving in gambling disorder and cocaine use disorder. Dialogues Clin. Neuro.

[15] Wu H., Zhou C., Guan X., Bai X., Guo T., Wu J., Chen J., Wen J., Wu C., Cao Z. (2023). Functional connectomes of akinetic-rigid and tremor within drug-naïve Parkinson’s disease. CNS Neurosci. Ther..

[16] Tong T.T., Vaidya J.G., Kramer J.R., Kuperman S., Langbehn D.R., O’Leary D.S. (2021). Impact of binge drinking during college on resting state functional connectivity. Drug Alcohol. Depend..

[17] Dukart J., Holiga S., Rullmann M., Lanzenberger R., Hawkins P.C.T., Mehta M.A., Hesse S., Barthel H., Sabri O., Jech R. (2021). JuSpace: A tool for spatial correlation analyses of magnetic resonance imaging data with nuclear imaging derived neurotransmitter maps. Hum. Brain Mapp..

[18] Premi E., Dukart J., Mattioli I., Libri I., Pengo M., Gadola Y., Cotelli M., Manenti R., Binetti G., Gazzina S. (2023). Unravelling neurotransmitters impairment in primary progressive aphasias. Hum. Brain Mapp..

[19] Pengo M., Mattioli I., Cantoni V., Dukart J., Gasparotti R., Buratti E., Todd E.G., Bouzigues A., Cash D.M., Russell L.L. (2023). Early neurotransmitters changes in prodromal frontotemporal dementia: A GENFI study. Neurobiol. Dis..

[20] Chen J., Wei Y., Xue K., Han S., Wang C., Wen B., Cheng J. (2023). The interaction between first-episode drug-naïve schizophrenia and age based on gray matter volume and its molecular analysis: A multimodal magnetic resonance imaging study. Psychopharmacology.

[21] Ren J., Yan L., Zhou H., Pan C., Xue C., Wu J., Liu W. (2023). Unraveling neurotransmitter changes in de novo GBA-related and idiopathic Parkinson’s disease. Neurobiol. Dis..

[22] Fiore A., Preziosa P., Tedone N., Margoni M., Vizzino C., Mistri D., Gueye M., Rocca M.A., Filippi M. (2023). Correspondence among gray matter atrophy and atlas-based neurotransmitter maps is clinically relevant in multiple sclerosis. Mol. Psychiatry.

[23] Cui S., Jiang P., Cheng Y., Cai H., Zhu J., Yu Y. (2023). Molecular mechanisms underlying resting-state brain functional correlates of behavioral inhibition. Neuroimage.

[24] Hirjak D., Schmitgen M.M., Werler F., Wittemann M., Kubera K.M., Wolf N.D., Sambataro F., Calhoun V.D., Reith W., Wolf R.C. (2022). Multimodal MRI data fusion reveals distinct structural, functional and neurochemical correlates of heavy cannabis use. Addict. Biol..

[25] Tang C., Ren P., Ma K., Li S., Wang X., Guan Y., Zhou J., Li T., Liang X., Luan G. (2022). The correspondence between morphometric MRI and metabolic profile in Rasmussen’s encephalitis. Neuroimage-Clin..

[26] Dugre J.R., Potvin S. (2022). The origins of evil: From lesions to the functional architecture of the antisocial brain. Front. Psychiatry.

[27] Van Essen D.C., Ugurbil K., Auerbach E., Barch D., Behrens T.E.J., Bucholz R., Chang A., Chen L., Corbetta M., Curtiss S.W. (2012). The Human Connectome Project: A data acquisition perspective. NeuroImage.

[28] Ide J.S., Li H.T., Chen Y., Le T.M., Li C.S.P., Zhornitsky S., Li C.R. (2020). Gray matter volumetric correlates of behavioral activation and inhibition system traits in children: An exploratory voxel-based morphometry study of the ABCD project data. Neuroimage.

[29] Li G., Zhang S., Le T.M., Tang X., Li C.-S.R. (2020). Neural Responses to Reward in a Gambling Task: Sex Differences and Individual Variation in Reward-Driven Impulsivity. Cereb. Cortex Commun..

[30] Li G., Chen Y., Wang W., Dhingra I., Zhornitsky S., Tang X., Li C.-S.R. (2020). Sex Differences in Neural Responses to the Perception of Social Interactions. Front. Hum. Neurosci..

[31] Li G., Le T.M., Wang W., Zhornitsky S., Chen Y., Chaudhary S., Zhu T., Zhang S., Bi J., Tang X. (2021). Perceived stress, self-efficacy, and the cerebral morphometric markers in binge-drinking young adults. NeuroImage Clin..

[32] Li G., Chen Y., Le T.M., Zhornitsky S., Wang W., Dhingra I., Zhang S., Tang X., Li C.-S.R. (2021). Perceived friendship and binge drinking in young adults: A study of the Human Connectome Project data. Drug Alcohol. Depend..

[33] Barch D.M., Burgess G.C., Harms M.P., Petersen S.E., Schlaggar B.L., Corbetta M., Glasser M.F., Curtiss S., Dixit S., Feldt C. (2013). Function in the human connectome: Task-fMRI and individual differences in behavior. Neuroimage.

[34] Li G., Li Y., Zhang Z., Chen Y., Li B., Hao D., Yang L., Yang Y., Li X., Li C.R. (2023). Sex differences in externalizing and internalizing traits and ventral striatal responses to monetary loss. J. Psychiatry Res..

[35] Li G., Zhang Z., Chen Y., Wang W., Bi J., Tang X., Li C.-S.R. (2022). Cognitive challenges are better in distinguishing binge from nonbinge drinkers: An exploratory deep-learning study of fMRI data of multiple behavioral tasks and resting state. J. Magn. Reson. Imaging.

[36] Li G., Chen Y., Chaudhary S., Li C.S., Hao D., Yang L., Li C.R. (2023). Sleep dysfunction mediates the relationship between hypothalamic-insula connectivity and anxiety-depression symptom severity bidirectionally in young adults. Neuroimage.

[37] Li G., Chen Y., Tang X., Li C.-S.R. (2021). Alcohol use severity and the neural correlates of the effects of sleep disturbance on sustained visual attention. J. Psychiatry Res..

[38] Li G., Zhong D., Li B., Chen Y., Yang L., Li C.R. (2023). Sleep Deficits Inter-Link Lower Basal Forebrain-Posterior Cingulate Connectivity and Perceived Stress and Anxiety Bidirectionally in Young Men. Int. J. Neuropsychopharmacol..

[39] Tzourio-Mazoyer N., Landeau B., Papathanassiou D., Crivello F., Etard O., Delcroix N., Mazoyer B., Joliot M. (2002). Automated anatomical labeling of activations in SPM using a macroscopic anatomical parcellation of the MNI MRI single-subject brain. Neuroimage.

[40] Shen X., Tokoglu F., Papademetris X., Constable R.T. (2013). Groupwise whole-brain parcellation from resting-state fMRI data for network node identification. Neuroimage.

[41] Hu S., Zhang S., Chao H.H., Krystal J.H., Li C.-S.R. (2016). Association of Drinking Problems and Duration of Alcohol Use to Inhibitory Control in Nondependent Young Adult Social Drinkers. Alcohol. Clin. Exp. Res..

[42] Hornoiu I.L., Lee A.M., Tan H., Nakovics H., Bach P., Mann K., Kiefer F., Sommer W.H., Vollstädt-Klein S. (2023). The Role of Unawareness, Volition, and Neural Hyperconnectivity in Alcohol Use Disorder: A Functional Magnetic Resonance Imaging Study. Biol. Psychiatry Cogn. Neurosci. Neuroimaging.

[43] Rapuano K.M., Rosenberg M.D., Maza M.T., Dennis N.J., Dorji M., Greene A.S., Horien C., Scheinost D., Todd Constable R., Casey B.J. (2021). Corrigendum to “Behavioral and brain signatures of substance use vulnerability in childhood” [Developmental Cognitive Neuroscience 46 (December) (2020) 100878]. Dev. Cogn. Neurosci..

[44] Yao G., Wei L., Jiang T., Dong H., Baeken C., Wu G.R. (2022). Neural mechanisms underlying empathy during alcohol abstinence: Evidence from connectome-based predictive modeling. Brain Imaging Behav..

[45] Lin X., Zhu X., Zhou W., Zhang Z., Li P., Dong G., Meng S., Deng J., Lu L. (2022). Connectome-based predictive modelling of smoking severity in smokers. Addict. Biol..

[46] Lichenstein S.D., Scheinost D., Potenza M.N., Carroll K.M., Yip S.W. (2021). Dissociable neural substrates of opioid and cocaine use identified via connectome-based modelling. Mol. Psychiatry.

[47] Yip S.W., Scheinost D., Potenza M.N., Carroll K.M. (2019). Connectome-Based Prediction of Cocaine Abstinence. Am. J. Psychiatry.

[48] Anton-Toro L.F., Shpakivska-Bilan D., Del Cerro-Leon A., Bruna R., Uceta M., Garcia-Moreno L.M., Maestu F. (2023). Longitudinal change of inhibitory control functional connectivity associated with the development of heavy alcohol drinking. Front. Psychol..

[49] Davies M. (2003). The role of GABAA receptors in mediating the effects of alcohol in the central nervous system. J. Psychiatry Neurosci. JPN.

[50] Maccioni P., Colombo G. (2019). Potential of GABA(B) Receptor Positive Allosteric Modulators in the Treatment of Alcohol Use Disorder. CNS Drugs.

[51] Logge W.B., Morley K.C., Haber P.S. (2022). GABA(B) Receptors and Alcohol Use Disorders: Clinical Studies. Curr. Top. Behav. Neurosci..

[52] Andersen K.A.A., Carhart-Harris R., Nutt D.J., Erritzoe D. (2021). Therapeutic effects of classic serotonergic psychedelics: A systematic review of modern-era clinical studies. Acta Psychiatry Scand..

[53] Dudek M., Hyytia P. (2016). Alcohol preference and consumption are controlled by the caudal linear nucleus in alcohol-preferring rats. Eur. J. Neurosci..

[54] Ashton M.K., Rueda A.V.L., Ho A.M., Noor Aizin N., Sharma H., Dodd P.R., Stadlin A., Camarini R. (2022). Sex differences in GABA(A) receptor subunit transcript expression are mediated by genotype in subjects with alcohol-related cirrhosis of the liver. Genes Brain Behav..

[55] Belmer A., Depoortere R., Beecher K., Newman-Tancredi A., Bartlett S.E. (2022). Neural serotonergic circuits for controlling long-term voluntary alcohol consumption in mice. Mol. Psychiatry.

[56] Belmer A., Patkar O.L., Lanoue V., Bartlett S.E. (2018). 5-HT1A receptor-dependent modulation of emotional and neurogenic deficits elicited by prolonged consumption of alcohol. Sci. Rep..

[57] McKenzie-Quirk S.D., Miczek K.A. (2003). 5-HT1A agonists: Alcohol drinking in rats and squirrel monkeys. Psychopharmacology.

[58] Hillmer A.T., Wooten D.W., Tudorascu D.L., Barnhart T.E., Ahlers E.O., Resch L.M., Larson J.A., Converse A.K., Moore C.F., Schneider M.L. (2014). The effects of chronic alcohol self-administration on serotonin-1A receptor binding in nonhuman primates. Drug Alcohol. Depend..

[59] Kim J., Zaso M.J., Desalu J.M., Park A. (2020). Interaction between the 5-hydroxytryptamine transporter-linked polymorphic region (5-HTTLPR) and negative life events in adolescent heavy drinking. J. Stud. Alcohol. Drugs.

[60] White A.M., Matthews D.B., Best P.J. (2000). Ethanol, memory, and hippocampal function: A review of recent findings. Hippocampus.

[61] Bliss T.V., Collingridge G.L. (1993). A synaptic model of memory: Long-term potentiation in the hippocampus. Nature.

[62] Morris R.G., Anderson E., Lynch G.S., Baudry M. (1986). Selective impairment of learning and blockade of long-term potentiation by an N-methyl-D-aspartate receptor antagonist, AP5. Nature.

[63] Baker T.E., Castellanos-Ryan N., Schumann G., Cattrell A., Flor H., Nees F., Banaschewski T., Bokde A., Whelan R., Buechel C. (2019). Modulation of orbitofrontal-striatal reward activity by dopaminergic functional polymorphisms contributes to a predisposition to alcohol misuse in early adolescence. Psychol. Med..

[64] Ridge J.P., Ho A.M.C., Dodd P.R. (2009). Sex Differences in NMDA Receptor Expression in Human Alcoholics. Alcohol Alcohol..

[65] Marron Fernandez de Velasco E., Tipps M.E., Haider B., Souders A., Aguado C., Rose T.R., Vo B.N., DeBaker M.C., Luján R., Wickman K. (2023). Ethanol-Induced Suppression of G Protein–Gated Inwardly Rectifying K^+^–Dependent Signaling in the Basal Amygdala. Biol. Psychiatry.

[66] Koob G.F., Weiss F. (1992). Neuropharmacology of Cocaine and Ethanol Dependence. Recent. Dev. Alcohol..

[67] Li T.K. (2000). Pharmacogenetics of responses to alcohol and genes that influence alcohol drinking. J. Stud. Alcohol..

[68] Grant K.A. (1994). Emerging neurochemical concepts in the actions of ethanol at ligand-gated ion channels. Behav. Pharmacol..

[69] Diana M., Brodie M., Muntoni A., Puddu M.C., Pillolla G., Steffensen S., Spiga S., Little H.J. (2003). Enduring Effects of Chronic Ethanol in the CNS: Basis for Alcoholism. Alcohol. Clin. Exp. Res..

[70] Olsen R.W. (2011). Extrasynaptic GABAA receptors in the nucleus accumbens are necessary for alcohol drinking. Proc. Natl. Acad. Sci. USA.

[71] Roh S., Matsushita S., Hara S., Maesato H., Matsui T., Suzuki G., Miyakawa T., Ramchandani V.A., Li T.-K., Higuchi S. (2011). Role of GABRA2 in Moderating Subjective Responses to Alcohol. Alcohol. Clin. Exp. Res..

[72] Duman R.S., Sanacora G., Krystal J.H. (2019). Altered Connectivity in Depression: GABA and Glutamate Neurotransmitter Deficits and Reversal by Novel Treatments. Neuron.

[73] Kraus C., Castrén E., Kasper S., Lanzenberger R. (2017). Serotonin and neuroplasticity–Links between molecular, functional and structural pathophysiology in depression. Neurosci. Biobehav. Rev..

[74] Akil H., Gordon J., Hen R., Javitch J., Mayberg H., McEwen B., Meaney M.J., Nestler E.J. (2018). Treatment resistant depression: A multi-scale, systems biology approach. Neurosci. Biobehav. Rev..

[75] Koohsari S., Sadabad F.E., Pittman B., Gallezot J.D., Carson R.E., van Dyck C.H., Li C.R., Potenza M.N., Matuskey D. (2023). Relationships of in vivo brain norepinephrine transporter and age, BMI, and gender. Synapse.

[76] Angarita G.A., Worhunsky P.D., Naganawa M., Toyonaga T., Nabulsi N.B., Li C.R., Esterlis I., Skosnik P.D., Radhakrishnan R., Pittman B. (2022). Lower prefrontal cortical synaptic vesicle binding in cocaine use disorder: An exploratory (11) C-UCB-J positron emission tomography study in humans. Addict. Biol..

[77] Li C.S., Potenza M.N., Lee D.E., Planeta B., Gallezot J.D., Labaree D., Henry S., Nabulsi N., Sinha R., Ding Y.S. (2014). Decreased norepinephrine transporter availability in obesity: Positron Emission Tomography imaging with (S,S)-[(11)C]O-methylreboxetine. Neuroimage.

[78] Zheng W., Li H., Cui B., Liang P., Wu Y., Han X., Li C.R., Li K., Wang Z. (2020). Altered multimodal magnetic resonance parameters of basal nucleus of Meynert in Alzheimer’s disease. Ann. Clin. Transl. Neurol..

[79] Li H., Jia X., Qi Z., Fan X., Ma T., Ni H., Li C.R., Li K. (2017). Altered Functional Connectivity of the Basal Nucleus of Meynert in Mild Cognitive Impairment: A Resting-State fMRI Study. Front. Aging Neurosci..

[80] Wang W., Zhornitsky S., Zhang S., Li C.R. (2021). Noradrenergic correlates of chronic cocaine craving: Neuromelanin and functional brain imaging. Neuropsychopharmacol. Off. Publ. Am. Coll. Neuropsychopharmacol..

[81] Peterson A.C., Zhang S., Hu S., Chao H.H., Li C.R. (2017). The Effects of Age, from Young to Middle Adulthood, and Gender on Resting State Functional Connectivity of the Dopaminergic Midbrain. Front. Hum. Neurosci..

[82] Zalesky A., Fornito A., Bullmore E.T. (2010). Network-based statistic: Identifying differences in brain networks. NeuroImage.

[83] Liu M., Zhang H., Shi F., Shen D. (2023). Hierarchical Graph Convolutional Network Built by Multiscale Atlases for Brain Disorder Diagnosis Using Functional Connectivity. IEEE Trans. Neural Networks Learn. Syst..

[84] Zhou W.R., Wang Y.M., Wang M., Wang Z.L., Zheng H., Wang M.J., Potenza M.N., Dong G.H. (2022). Connectome-based prediction of craving for gaming in internet gaming disorder. Addict. Biol..

[85] Baker A.L., Thornton L.K., Hiles S., Hides L., Lubman D.I. (2012). Psychological interventions for alcohol misuse among people with co-occurring depression or anxiety disorders: A systematic review. J. Affect. Disord..

[86] Debell F., Fear N.T., Head M., Batt-Rawden S., Greenberg N., Wessely S., Goodwin L. (2014). A systematic review of the comorbidity between PTSD and alcohol misuse. Soc. Psychiatry Psychiatry Epidemiol..

[87] Gazula H., Rootes-Murdy K., Holla B., Basodi S., Zhang Z., Verner E., Kelly R., Murthy P., Chakrabarti A., Basu D. (2023). Federated Analysis in COINSTAC Reveals Functional Network Connectivity and Spectral Links to Smoking and Alcohol Consumption in Nearly 2000 Adolescent Brains. Neuroinform.

[88] Galinowski A., Miranda R., Lemaitre H., Artiges E., Paillère Martinot M.L., Filippi I., Penttilä J., Grimmer Y., Noort B.M., Stringaris A. (2020). Heavy drinking in adolescents is associated with change in brainstem microstructure and reward sensitivity. Addict. Biol..

[89] Harper J., Malone S.M., Wilson S., Hunt R.H., Thomas K.M., Iacono W.G. (2021). The Effects of Alcohol and Cannabis Use on the Cortical Thickness of Cognitive Control and Salience Brain Networks in Emerging Adulthood: A Co-twin Control Study. Biol. Psychiatry.

[90] Logtenberg E., Overbeek M.F., Pasman J.A., Abdellaoui A., Luijten M., van Holst R.J., Vink J.M., Denys D., Medland S.E., Verweij K.J.H. (2022). Investigating the causal nature of the relationship of subcortical brain volume with smoking and alcohol use. Br. J. Psychiatry.

[91] Rane R.P., de Man E.F., Kim J., Goergen K., Tschorn M., Rapp M.A., Banaschewski T., Bokde A.L.W., Desrivieres S., Flor H. (2022). Structural differences in adolescent brainscan predict alcohol misuse. Elife.

[92] Wang W., Zhornitsky S., Le T.M., Zhang S., Li C.-S.R. (2020). Heart Rate Variability, Cue-Evoked Ventromedial Prefrontal Cortical Response, and Problem Alcohol Use in Adult Drinkers. Biol. Psychiatry Cogn. Neurosci. Neuroimaging.

[93] Zhang S., Zhornitsky S., Le T.M., Li C.R. (2019). Hypothalamic Responses to Cocaine and Food Cues in Individuals with Cocaine Dependence. Int. J. Neuropsychopharmacol..

[94] Zhornitsky S., Zhang S., Ide J.S., Chao H.H., Wang W., Le T.M., Leeman R.F., Bi J., Krystal J.H., Li C.R. (2019). Alcohol Expectancy and Cerebral Responses to Cue-Elicited Craving in Adult Nondependent Drinkers. Biol. Psychiatry Cogn. Neurosci. Neuroimaging.

