# Supplementary material for: Functional Networks of Reward and Punishment Processing and Their Molecular Profiles Predicting the Severity of Young Adult Drinking
Source: Brain Sci. 2024 Jun 18;14(6):610. doi: 10.3390/brainsci14060610 (PMC11201596; doi:10.3390/brainsci14060610)
Supplement: Supplementary file 1 [file brainsci-14-00610-s001.zip › brainsci-3017488-supplementary.pdf]

Supplement:

Li et al., Functional networks of reward and punishment processing and their molecular profiles predicting the severity of young adult drinking

Supplementary Methods

Imaging protocol, behavioral tasks, and data preprocessing

MRI was done using a customized 3 T Siemens Connectome Skyra with a standard 32-channel Siemens receiver head coil and a body transmission coil. T1-weighted high-resolution structural images were acquired using a 3D MPRAGE sequence with 0.7 mm isotropic resolution (FOV = 224 × 224 mm, matrix = 320 × 320, 256 sagittal slices, TR = 2400 ms, TE = 2.14 ms, TI = 1000 ms, FA = 8°) and used to register functional MRI data to a standard brain space. FMRI data were collected using gradient-echo echo-planar imaging (EPI) with 2.0 mm isotropic resolution (FOV = 208 × 180 mm, matrix = 104 × 90, 72 slices, TR = 720 ms, TE = 33.1 ms, FA = 52°, multi-band factor = 8).

Participants completed two runs of a gambling task each with 4 blocks (~3 m and 12 s each run) – 2 of punishment and 2 of reward – in a fixed order (run 1: punishment – reward – punishment – reward; and run 2: reward – punishment – punishment – reward) with a fixation period (15 s) between blocks. The participants guessed whether the number of a mystery card (represented by a '?' and ranging from 1 to 9) was larger or smaller than 5 by pressing a corresponding button (Barch et al., 2013). The feedbacks comprised a green up-pointing arrow for correct guess and $1 win, a red down-pointing arrow for $0.5 loss; or a gray double-headed arrow for a wash (mystery card number = 5). The mystery number was controlled by the program and shown for 1.5 s, followed by the feedback for 1.0 s. There was a 1.0 s inter-trial interval with a "+" shown on the screen. Each block contained 8 trials. In reward blocks, 6 win trials were pseudo-randomly interleaved with either 1 neutral and 1 loss trial, 2 neutral trials, or 2 loss trials. In punishment blocks, 6 loss trials were interleaved

with either 1 neutral and 1 win trial, 2 neutral trials, or 2 win trials. Thus, the amount of money won was the same across subjects.

BOLD data were analyzed with Statistical Parametric Mapping (SPM8, Welcome Department of Imaging Neuroscience, University College London, U.K.), following our published routines (Wang et al., 2020; Zhang et al., 2019; Zhornitsky et al., 2019). Images of each individual subject were first realigned (motion corrected). A mean functional image volume was constructed for each subject per run from the realigned image volumes. These mean images were co-registered with the high-resolution structural MPRAGE image and then segmented for normalization with affine registration followed by nonlinear transformation. The normalization parameters determined for the structural volume were then applied to the corresponding functional image volumes for each subject. The voxel is of 2x2x2 mm^3 after spatial normalization. Finally, the images were smoothed with a Gaussian kernel of 4 mm at Full Width at Half Maximum.

The GLM and 2nd-level analyses

Briefly, a statistical analytical block design was constructed for each individual subject, using a general linear model (GLM) by convolving the canonical hemodynamic response function (HRF) with a boxcar function in SPM. Realignment parameters in all six dimensions were entered in the model as covariates. We constructed for each individual subject the statistical contrast "reward vs. baseline", "punishment vs. baseline" and "reward vs. punishment", with baseline = 15-s fixation period between blocks in gambling task.

Functional connectivity and connectome-based predictive modeling (CPM)

CPM took group connectivity matrices and behavioral data (in this case PC1) as inputs to generate a predictive model of PC1 from connectivity matrices. Edges and PC1 from the training dataset were correlated using regression analyses to identify positive and negative predictive networks.

Positive and negative networks were networks for which higher and lower edge weights (connectivity), respectively, were associated with PC1. While both networks were used for predicting PC1, they were by definition independent as a single edge could not be both a positive and negative predictor. Single subject summary statistics were then created as the sum of the significant edge weights in each network and entered into predictive models assuming linear relationships with PC1. The resultant polynomial coefficients (linear equation including slope and intercept) were then applied to the test dataset to predict PC1. We employed leave-one-out cross-validation, where a single "left-out" participant's predicted value was generated by taking the data from all other participants as the training dataset in an iterative manner until all participants had a predicted value. Model performance (i.e., correspondence between predicted and actual values) was assessed using Spearman's rho correlations. In leave-one-out cross-validation, analyses in the leave-one-out folds were not truly independent and the number of degrees of freedom was thus overestimated for parametric p-values of correlation. Instead of parametric testing, we therefore performed permutation testing. To generate the null distributions for significance testing, we randomly shuffled the correspondence between PC1's and connectivity matrices 1,000 times and re-ran the CPM analysis with the shuffled data. Based on the null distributions, the p-values for leave-one-out predictions were calculated.

Supplementary Results

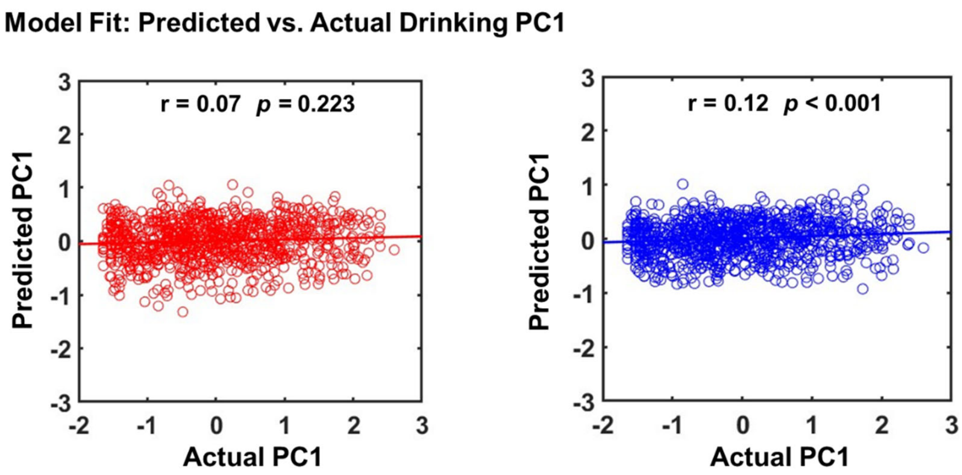

Supplementary Figure S1. Macroscale network connectivities of loss processing in predicting drinking PC1. The correlation between actual (x-axis) and predicted (y-axis) drinking PC1 values generated using CPM with a five-fold cross-validation.

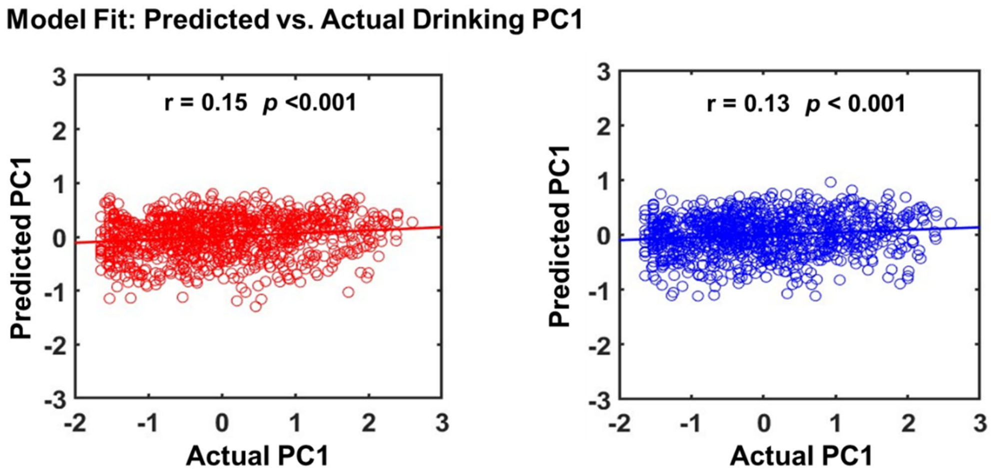

Supplementary Figure S2. Macroscale network connectivities of win processing in predicting drinking PC1. The correlation between actual (x-axis) and predicted (y-axis) drinking PC1 values generated using CPM with a five-fold cross-validation.

Supplementary References

Barch, D.M., Burgess, G.C., Harms, M.P., Petersen, S.E., Schlaggar, B.L., Corbetta, M., Glasser, M.F., Curtiss, S., Dixit, S., Feldt, C., Nolan, D., Bryant, E., Hartley, T., Footer, O., Bjork, J.M., Poldrack, R., Smith, S., Johansen-Berg, H., Snyder, A.Z., Van Essen, D.C., Consortium, W.U.-M.H., 2013. Function in the human connectome: task-fMRI and individual differences in behavior. Neuroimage 80, 169-189.

Wang, W., Zhornitsky, S., Le, T.M., Zhang, S., Li, C.-S.R., 2020. Heart Rate Variability, Cue-Evoked Ventromedial Prefrontal Cortical Response, and Problem Alcohol Use in Adult Drinkers. Biological

Psychiatry: Cognitive Neuroscience and Neuroimaging 5, 619-628.

Zhang, S., Zhornitsky, S., Le, T.M., Li, C.R., 2019. Hypothalamic Responses to Cocaine and Food Cues in Individuals with Cocaine Dependence. Int J Neuropsychopharmacol 22, 754-764.

Zhornitsky, S., Zhang, S., Ide, J.S., Chao, H.H., Wang, W., Le, T.M., Leeman, R.F., Bi, J., Krystal, J.H., Li, C.R., 2019. Alcohol Expectancy and Cerebral Responses to Cue-Elicited Craving in Adult Nondependent Drinkers. Biol Psychiatry Cogn Neurosci Neuroimaging 4, 493-504.
